# Synergistic effects of multi-strain probiotics and Chinese herbal medicine on growth performance and gut health in weaned piglets

**DOI:** 10.3389/fvets.2025.1677127

**Published:** 2025-10-01

**Authors:** Kai Ji, Sheng Lei, Lingling Wang, Lang Tian, Zizeng Wen, Rongjing Shi, Lin Liu, Jinjia Liu, Baoming Shi, Jian Wang

**Affiliations:** ^1^Animal Husbandry and Fisheries Research Center of Guangdong Haid Group Co., Ltd., Guangzhou, China; ^2^Animal Science and Technology College, Northeast Agricultural University, Harbin, China; ^3^Department of Basic Medical Sciences, Changzhi Medical College, Changzhi, China

**Keywords:** Chinese herbal medicine, probiotic, weaned piglets, growth performance, microbiome, metabolome

## Abstract

**Background:**

Weaning stress represents a considerable challenge in global swine production. While probiotics and Chinese herbal medicine have been extensively studied as individual interventions, their combined application as alternative feed additives in swine production requires further investigation.

**Methods:**

Forty-five weaned piglets (35 ± 3 days old) were randomly allocated to five treatment groups (*n* = 9 per group) for a 28-day feeding trial: control (CON), antibiotic (A), probiotic (PRO), Chinese herbal medicine (CHM), and probiotic plus Chinese herbal medicine (PROC). Growth performance, serum antioxidant levels, and immune parameters were assessed alongside 16S rRNA microbiome sequencing and liquid chromatography-mass spectrometry metabolomics analysis.

**Results:**

The PROC group significantly improved growth performance compared to controls (*p* < 0.05), showing 8.91% higher final body weight, significantly increased average daily gain, and the most efficient feed conversion ratio (1.55) among treatments. Serum analysis indicated a significant increase in total antioxidant capacity (T-AOC) in the PROC group relative to the other groups. The probiotic (PRO) and PROC groups enhanced superoxide dismutase (SOD) and glutathione peroxidase (GSH-Px) activities, while reducing malondialdehyde (MDA) levels (*p* < 0.05). Pro-inflammatory cytokines IL-2 and IL-6 were significantly reduced in the PRO and PROC groups, while immunoglobulins IgA and IgG levels were increased (*p* < 0.05). Microbiota analysis revealed increased *α*-diversity (Shannon and Chao1 indices) and altered community structure in the PROC group. Metabolomic profiling identified 5,090 metabolites with distinct profiles between groups based on OPLS-DA. KEGG pathway analysis indicated that the PRO group exhibited enrichment in nucleotide and purine metabolism, whereas the PROC group activated supplementary pathways, including purine and lipid metabolism.

**Conclusion:**

These findings suggest that combined probiotic and Chinese herbal medicine supplementation may serve as an effective feed strategy for promoting intestinal health and alleviating weaning stress, providing valuable insights for developing antibiotic alternatives in swine production.

## Introduction

Swine production plays a pivotal role in global food security. As the world’s leading producer, China produces over 57 million tons of pork annually, as reported by the National Bureau of Statistics of China in 2024. Although modern swine production has achieved remarkable efficiency, weaning stress remains a significant challenge for producers globally. The weaning transition subjects piglets to nutritional, environmental, and microbial stresses. These stresses cause appetite suppression, diarrhea, growth retardation, and even elevated mortality rates (1). In recent decades, antibiotics have been widely used in piglet production. Historically, antibiotics functioned as the cornerstone strategy for managing post-weaning complications through pathogen suppression and microbiome modulation (2). However, increasing evidence of antibiotic resistance prompted regulatory changes, culminating in China’s prohibition of antibiotic feed additives in 2020. This regulatory shift necessitated the development of alternative intervention strategies.

The search for effective alternatives has focused on several approaches, including Zinc oxide (3), organic acids (4), Chinese herbal medicine (5), and probiotics (6), each demonstrating varying degrees of efficacy in alleviating weaning stress. Zinc oxide and organic acids show limited efficacy and potential adverse effects including environmental concerns, mineral metabolism disorders, and gastrointestinal irritation (7–10). In contrast, Chinese herbal medicine and probiotics offer greater biosafety and sustainability. Many Chinese herbal medicines demonstrate broad-spectrum antimicrobial properties through key bioactive compounds including alkaloids, flavonoids, and polyphenols, which inhibit both gram-positive and gram-negative bacteria (11). These compounds also modulate immune responses, improve digestive efficiency, and promote nutrient absorption (12, 13). Probiotics achieve therapeutic effects through intestinal colonization and subsequent establishment of beneficial microbial dominance. These microorganisms produce various antimicrobial substances, including bacteriocins, organic acids, and antimicrobial peptides, that competitively inhibit pathogenic bacteria. This biological mechanism represents a milder and more sustained approach than chemical inhibition methods (14, 15).

Chinese herbal medicine has been utilized in livestock production for over 2000 years (16). Recent studies demonstrate that treatments with Chinese herbal medicines can effectively alleviate weaning stress in piglets. Several studies have documented specific benefits of herbal interventions. One study evaluated a combination of buckwheat, thyme, turmeric, black pepper, and ginger in pig feeding (17). This dietary supplement enhanced growth performance and elevated various blood cell populations, including lymphocytes, red blood cells, and white blood cells. Another study investigated extracts from Scutellaria baicalensis and *Lonicera japonica*, collectively referred to as SLE (18). The treatment of SLE improved immune function in both sows and their nursing piglets. The treatment resulted in elevated levels of immunoglobulins (IgA and IgG) and IL-10 cytokine in the serum and milk of sows. Furthermore, SLE enhanced reproductive performance in the sows. A recent study demonstrated that Qi-Zhu-Gui-Shao powder (QZGSP), when used as a feed additive, enhanced antioxidant capacity and altered the fecal microbiota in both sows and their offspring (19). These studies indicate that Chinese herbal medicine can enhance pig health when incorporated as feed additives. The treatments demonstrated beneficial effects on growth performance, immune function, and metabolic processes in both sows and their progeny.

In addition to Chinese herbal medicine, probiotics are effective as feed supplements in livestock production. Various probiotics, including Lactobacillus (20), Bacillus (21), Saccharomyces (22), and Clostridium (23), have been employed as feed supplements. However, single probiotic strains often exhibit limited functional diversity. The combination of multiple probiotics will enhance their application potential in livestock feeding. Research indicated that incorporating a multiple-strain Lactobacillus mixture as a feed supplement markedly enhances daily feed intake and body weight gain in weaned piglets, while also significantly decreasing the occurrence of diarrhea (24). Wang et al. demonstrated that a two-strain Bacillus mixture improved the intestinal immune function of weaned piglets by altering the composition of intestinal flora and the levels of microbial metabolites (25). The concurrent application of *Lactobacillus plantarum* QP28-1a and *Bacillus subtilis* QB8a preserved an acidic intestinal milieu in weaned piglets, thereby suppressing pathogen proliferation and mitigating intestinal inflammation (26). Research demonstrates that multi-strain probiotic combinations enhance intestinal immunity and nutrient digestibility in weaned piglets more effectively than single-strain applications.

While previous studies demonstrate that Chinese herbal medicine and probiotics individually improve health outcomes in weaned piglets, each approach has specific limitations when applied individually (27). Chinese herbal medicine provides multi-target therapeutic effects but with slow onset and limited bioavailability, whereas probiotics face challenges with inconsistent colonization efficiency and individual variability (28, 29). Theoretically, Chinese herbal medicine can serve as nutritional substrates for probiotics, thereby promoting beneficial bacterial proliferation. Concurrently, probiotics may facilitate the metabolic transformation of herbal constituents, leading to enhanced bioavailability. Research indicates that the combined application of probiotics and Chinese herbal medicine improves dietary palatability, decreases diarrhea incidence, enhances immune function and antioxidant capacity, and promotes growth and development in weaned piglets (12, 30). Systematic investigations into the collaborative mechanisms between Chinese herbal medicine and probiotics in weaned piglets are limited. This research investigated the impact of a combination of three Chinese herbs and five probiotic strains on growth performance, antioxidant capacity, and immune function in weaned piglets. We analyzed alterations in fecal microbiota composition and metabolomic profiles. This research aims to clarify the synergistic mechanisms and functional patterns of Chinese herbal medicine and probiotics in weaned piglets, providing a theoretical foundation for precision management and scientific feeding practices in weaned piglet production.

## Materials and methods

### Probiotic strains, Chinese herbal medicine and feed

The probiotic strains (*E. faecalis*, *L. plantarum*, *P. pentosaceus*, *B. coagulans*, and *S. boulardii*) used for fermented feed were isolated, screened, identified, and preserved by the Guangdong HAID Research Institute. After two days (48 h) of liquid fermentation at room temperature (25 °C), the viable counts of the five probiotics were as follows: *L. plantarum* reached approximately 4 × 10^9^ CFU/mL, *P. pentosaceus* reached approximately 3 × 10^9^ CFU/mL, *E. faecalis* attained approximately 1 × 10^9^ CFU/mL, *B. coagulans* achieved approximately 2 × 10^9^ CFU/mL, and *S. boulardii* reached approximately 3 × 10^8^ CFU/mL. This study employed a Chinese herbal medicine formula comprising *Astragalus membranaceus* (Huang Qi), *Magnolia officinalis* (Hou Po), and *Codonopsis pilosula* (Dang Shen) in a 1:1:1 ratio (powdered form to ensure uniform mixing). The basal feed was developed in compliance with the Chinese NY/T 65–2004 standard for swine nutrition. The basal feed comprised 54.32% corn, 15.12% wheat bran, 11.33% soybean meal, along with additional components detailed in Table 1. Probiotics were incorporated into the feed through mixing 5 kg of fermentation broth with 15 kg of water, which was then thoroughly mixed with 80 kg of the basal diet. Chinese herbal medicine and doxycycline hydrochloride were included in the basal diet at a concentration of 1 g/kg.

**Table 1:** Composition and nutrient levels of the basal diet used in the weaned piglet feeding trial (air-dried basis).

Items	Contents (%)	Nutritional components	Content (%)
Corn	54.32	Crude protein	20.85
Wheat bran	15.12	Crude fiber	3.28
Soybean meal	11.33	Crude ash	6.45
Fish meal	4.00	Calcium	0.85
Whey powder	6.00	Available phosphorus	0.42
Soybean oil	3.50	Total phosphorus	0.68
Stone powder	1.20	Lysine	1.35
Calcium hydrogen phosphate	1.80	Methionine + Cystine	0.72
Salt	0.35	Threonine	0.85
L-Lysine·HCl	0.45	Tryptophan	0.23
DL-Methionine	0.18		
L-Threonine	0.15		
L-Tryptophan	0.05		
Choline chloride	0.05		
Premix^a^	1.50		
Total	100.00		

a Premix for each kg of feed provides: vitamin A 12,000 IU, vitamin D₃ 2,500 IU, vitamin E 40 IU, vitamin K₃ 3.0 mg, vitamin B₁ 2.0 mg, vitamin B₂ 6.0 mg, vitamin B₆ 4.0 mg, vitamin B₁₂ 0.025 mg, niacin 35.0 mg, pantothenic acid 15.0 mg, folic acid 1.5 mg, biotin 0.2 mg, iron 100 mg, copper 150 mg, zinc 100 mg, manganese 50 mg, iodine 0.5 mg, selenium 0.3 mg.

### Animals and study design

A total of 45 healthy weaned crossbred piglets (Duroc × Landrace × Yorkshire, 35 ± 3 days of age, initial body weight 9.62 ± 0.23 kg) were obtained from Haid Pig Breeding Base in Heshan, Guangdong Province, China, and were randomly assigned to five treatment groups (*n* = 9 per group), ensuring comparable body weights and equal sex distribution. Each group comprised three biological replicates, with three weaned piglets in each replicate. All piglets were housed under controlled environmental conditions: temperature 22–28 °C, relative humidity 60–80%, ventilation rate 15–20 air changes per hour, and a 12:12 h light–dark cycle (lights on 06:00–18:00). Each replicate of three weaned piglets was housed in individual pens measuring 2.0 m × 1.5 m × 1.2 m, which included slatted flooring, automatic feeders, and nipple drinkers. The experimental design included five treatment groups: (1) CON (control): basal diet; (2) A (antibiotic): basal diet + doxycycline hydrochloride; (3) PRO (probiotic): basal diet + probiotic fermentation broth; (4) CHM (Chinese herbal medicine): basal diet + Chinese herbal medicine formula; (5) PROC (probiotic and Chinese herbal medicine): basal diet + probiotic fermentation broth and Chinese herbal medicine. Before the formal feeding trial, all weaned piglets underwent a 7-day standardized adaptation period with a starter diet to mitigate weaning stress. A 28-day formal feeding phase was subsequently conducted utilizing automated feeding systems. During the experimental period, piglets were provided with unlimited access to feed and water, and daily feed intake was systematically documented. All weaned piglets underwent standard management procedures, which included ear tagging for identification, routine vaccinations, and scheduled deworming treatments. Pens underwent daily cleaning, and facilities were disinfected biweekly to maintain hygiene standards.

### Analysis of probiotic fermentation broth

The pH of fermentation broth samples was determined using a calibrated pH meter (FE28, Mettler Toledo). Organic acids were quantified using HPLC (Agilent 1,260 Infinity II) with an Aminex HPX-87H ion-exclusion column (Bio-Rad). *β*-Glucan content was assessed utilizing a commercial enzymatic assay kit (Megazyme, Wicklow, Ireland). Total protein content was determined using the Kjeldahl method according to AOAC Official Method 2001.11 (Association of Official Analytical Chemists International, 2005). The analysis was performed using a Kjeltec 8,400 analyzer (FOSS Analytical, Hillerød, Denmark).

### Growth performance

Body weight and feed intake were assessed weekly during the experimental period. The measurements for average daily gain (ADG), average daily feed intake (ADFI), and feed conversion ratio (FCR) were derived from the initial body weight (IBW, kg), final body weight (FBW, kg), and total feed intake (TFI, kg) documented throughout the study. The feed conversion ratio was calculated using the formula FCR = TFI/(FBW−IBW), allowing for an evaluation of the efficiency with which the animals converted feed into body weight among various treatment groups.

### Sample collection

On day 28, six piglets with similar body weights were selected from each group for blood and fecal sample collection. Blood samples were collected via auricular venipuncture, allowed to clot at room temperature for 30 min, then centrifuged at 1600 × g for 30 min at 4 °C. The serum was aliquoted into cryovials and stored at −80 °C for subsequent biochemical analysis. Fecal samples were obtained from each piglet and placed in sterile collection tubes, then stored at −80 °C for later analysis.

### Serum biochemical analysis

Serum antioxidant parameters, including diamine oxidase (DAO), superoxide dismutase (SOD), glutathione (GSH), glutathione peroxidase (GSH-PX), malondialdehyde (MDA), catalase (CAT), and total antioxidant capacity (T-AOC), were assessed using commercially available assay kits from Jiangsu Meimian Industrial Co., Ltd., China, in accordance with the manufacturer’s protocols. All samples were measured in triplicate to ensure accuracy and precision. Serum concentrations of cytokines, including interferon-gamma (IFN-*γ*), tumor necrosis factor-alpha (TNF-*α*), interleukins (IL-1β, IL-2, IL-6, IL-10, and IL-12), as well as immunoglobulins (IgA, IgG, IgM), were quantified using enzyme-linked immunosorbent assay (ELISA) kits from Jiangsu Meimian Industrial Co., Ltd., China, following the manufacturer’s protocols. All ELISA measurements were performed in triplicate, and standard curves were generated using serial dilutions of known standards provided with each kit to ensure accurate quantification of target analytes.

### 16S rRNA sequencing and analysis

Using previously stored fecal samples kept at −80 °C as test subjects, 16S rDNA sequencing and analysis were performed following the protocols described in the earlier research of Peiffer et al. (31). In brief, total bacterial DNA from the feces was extracted using a QIAamp PowerFecal Pro DNA Kit (Qiagen, Germany). The V3-V4 variable region of 16S rRNA was PCR-amplified using a pair of universal primers 515F-806R (32). After purification and quality control, 16S rRNA amplicons were prepared for library construction through end repair, A-tailing, and addition of sequencing adapters. Sequencing was conducted on the Illumina HiSeq2500 PE250 platform (Illumina, San Diego, CA, United States) by Novogene Bioinformatics Technology Co., Ltd. in Beijing, China.

Paired-end reads were merged utilizing FLASH (v1.2.11) and subsequently underwent quality control through QIIME2 (v2023.5). The research employed DADA2 in conjunction with QIIME2 to denoise sequences and produce Amplicon Sequence Variants (ASVs), subsequently constructing an ASV table. Species were annotated according to ASVs utilizing the SILVA (v138) reference database in QIIME2. Alpha and beta diversity of fecal microbiota were assessed utilizing QIIME2. The microbiomeMarker (v1.5.2) R package facilitated the execution of Linear Discriminant Analysis Effect Size (LEfSe) to identify and differentiate specific biomarkers across groups.

### Fecal metabolomics analysis

Fecal samples were sent to Wuhan Metware Biotechnology Co., Ltd. for untargeted metabolomics analysis via liquid chromatography-mass spectrometry (Thermo Fisher, Dreieich, Germany). Sample aliquots of 20 mg were extracted with 400 μL of 70% methanol. After sonication and centrifugation, the supernatants were collected for LC–MS analysis. Chromatographic separation was performed on a Waters ACQUITY Premier HSS T3 column (1.8 μm, 2.1 mm × 100 mm). Mobile phase A consisted of 0.1% formic acid in water, and mobile phase B was 0.1% formic acid in acetonitrile. The column temperature was maintained at 40 °C, with a flow rate of 0.4 mL/min and an injection volume of 4 μL. Raw data were transformed into mzML format. Peak extraction and alignment were performed using XCMS. Peaks exhibiting missing rates exceeding 50% were excluded from the analysis. Missing values were imputed utilizing KNN and the one-fifth minimum value methods. Peak areas were adjusted utilizing the SVR algorithm. Metabolite identification was conducted through searches in in-house, public, and predicted databases. Metabolites with identification scores >0.5 and QC sample coefficients of variation <0.3 were retained for subsequent analysis.

Orthogonal partial least squares discriminant analysis (OPLS-DA) was performed using the OPLSR. Anal function in the MetaboAnalyst R package of R software. Volcano plots were generated using ggplot2 in R to filter metabolites based on log₂ (fold change) and −log₁_0_ (*p*-value). *p*-values were calculated through statistical tests. Variable importance in projection (VIP) scores were obtained using OPLS-DA dimensionality reduction. Metabolites with *p*-values <0.05 and VIP scores >1 were considered statistically significant. Functional analysis of differential metabolites was conducted. Kyoto Encyclopedia of Genes and Genomes (KEGG) enrichment analysis was performed to identify significantly enriched metabolic pathways.

### Statistical analysis

Data were analyzed using one-way ANOVA (SPSS 20.0, SPSS Inc., Chicago, IL, United States), followed by Tukey’s HSD post-hoc test for multiple comparisons when significant differences were detected. Data are presented as mean ± standard deviation (SD), with statistical significance set at *p* < 0.05. Histograms were created utilizing GraphPad Prism 10 software (GraphPad Prism Inc., United States). Spearman correlation analysis was conducted to examine the relationships between fecal microbial communities and metabolites. The pheatmap package in R software was utilized for visualizing the results.

## Results

### Analysis of physicochemical properties and composition of single-strain and multi-strain fermentation broths

The physicochemical properties and composition of single-strain and multi-strain fermentation broths are presented in Table 2. Multi-strain fermentation showed significantly lower pH values (3.887 vs. 4.077–4.343) and higher organic acid content than single-strain fermentation, indicating enhanced acid production capacity. Furthermore, multi-strain fermentation broth resulted in increased levels of protein (crude protein) and polysaccharide (Beta-glucan), thereby supplying additional nutrients for intestinal epithelial cells.

**Table 2:** Physicochemical characteristics and nutrient composition of single-strain and multi-strain fermentation broths.

Item	*E. faecalis*	*L. plantarum*	*P. pentosaceus*	*B. coagulans*	*S. boulardii*	Multi-strain
pH	4.077 ± 0.033^b^	4.153 ± 0.017^b^	4.303 ± 0.045^a^	4.180 ± 0.043^b^	4.343 ± 0.037^a^	3.887 ± 0.061^c^
Lactic acid (mg/mL)	15.633 ± 0.246^b^	15.117 ± 0.425^b^	13.710 ± 0.311^c^	14.383 ± 0.641^c^	10.630 ± 0.361^d^	16.877 ± 0.577^a^
acetic acid (mg/mL)	2.840 ± 0.071^d^	3.113 ± 0.012^c^	3.540 ± 0.086^b^	2.417 ± 0.041^e^	3.790 ± 0.118^b^	4.230 ± 0.205^a^
butyric acid (mg/mL)	0.723 ± 0.074^d^	0.613 ± 0.019^d^	0.973 ± 0.045^c^	1.323 ± 0.054^b^	0.810 ± 0.022^c^	1.613 ± 0.063^a^
Succinic acid (mg/mL)	1.400 ± 0.062^d^	1.327 ± 0.012^d^	1.473 ± 0.045^c^	1.657 ± 0.088^b^	1.690 ± 0.049^b^	1.920 ± 0.037^a^
Oxalic acid (mg/mL)	0.437 ± 0.012^a^	0.423 ± 0.017^a^	0.380 ± 0.008^b^	0.353 ± 0.005^c^	0.330 ± 0.008^c^	0.317 ± 0.017^c^
Beta-glucan (mg/mL)	0.072 ± 0.005^e^	0.076 ± 0.014^d^	0.079 ± 0.022^d^	0.096 ± 0.029^c^	0.102 ± 0.017^b^	0.118 ± 0.014^a^
Crude protein (mg/mL)	15.927 ± 0.323^d^	15.640 ± 0.246^d^	14.390 ± 0.155^e^	17.257 ± 0.083^c^	17.943 ± 0.563^b^	19.920 ± 0.257^a^

Data are presented as mean ± standard error. Different lowercase letters in the same row indicate significant differences (*p* < 0.05).

### Effects of probiotics and Chinese herbal medicine supplementation on growth performance in weaned piglets

**Figure 1:**
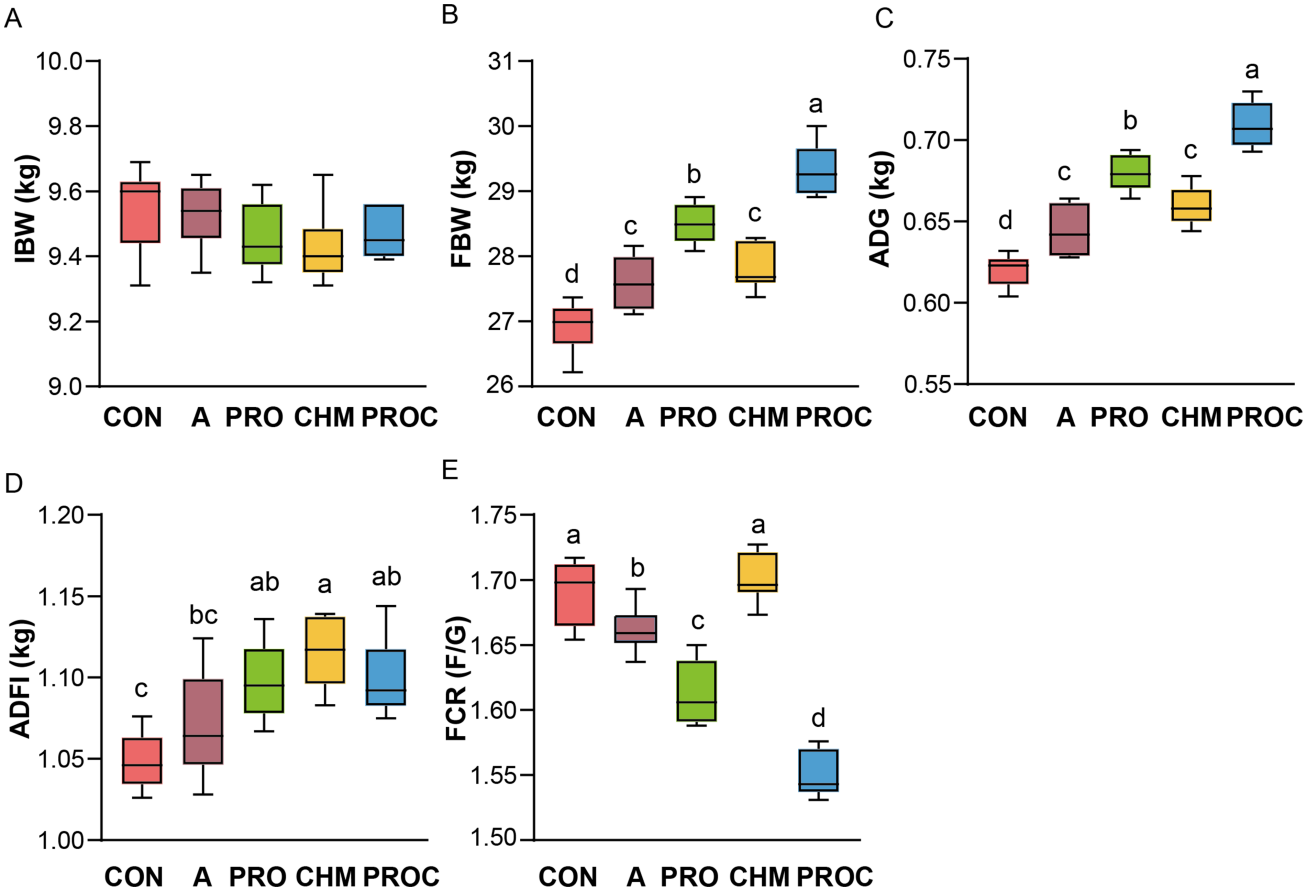
Comparison of growth performance of weaned piglets in different treatment groups. **(A)** initial body weight (IBW); **(B)** Final body weight (FBW); **(C)** average day gain (ADG); **(D)** average daily feed intake (ADFI); **(E)** feed conversion ratio (FCR). Data are presented as mean ± SD (*n* = 9 per group). Different letters indicate significant differences among groups determined by one-way ANOVA followed by Tukey’s HSD post-hoc test for multiple comparisons (*p* < 0.05).

Growth performance of weaned piglets was evaluated following probiotic and Chinese herbal medicine supplementation. Although initial body weights were similar across all treatment groups (Figure 1A). After 28 days, the PRO and PROC groups showed 5.89 and 8.91% increases in final body weight, respectively, compared to the control group (*p* < 0.05; Figure 1B). The average daily gain was significantly higher in the PRO and PROC groups than in the control group (*p* < 0.05, Figure 1C). Average daily feed intake showed a significant increase in the PRO, CHM, and PROC groups by 4.69, 6.35, and 4.94%, respectively, when compared to the control group (*p* < 0.05, Figure 1D). The feed conversion ratio was significantly lower in the CON and CHM groups compared to the other groups (*p* < 0.05, Figure 1E). The PROC group recorded the lowest feed conversion ratio at 1.55.

### Serum antioxidant enzyme activities and oxidative stress markers in weaned piglets

**Figure 2:**
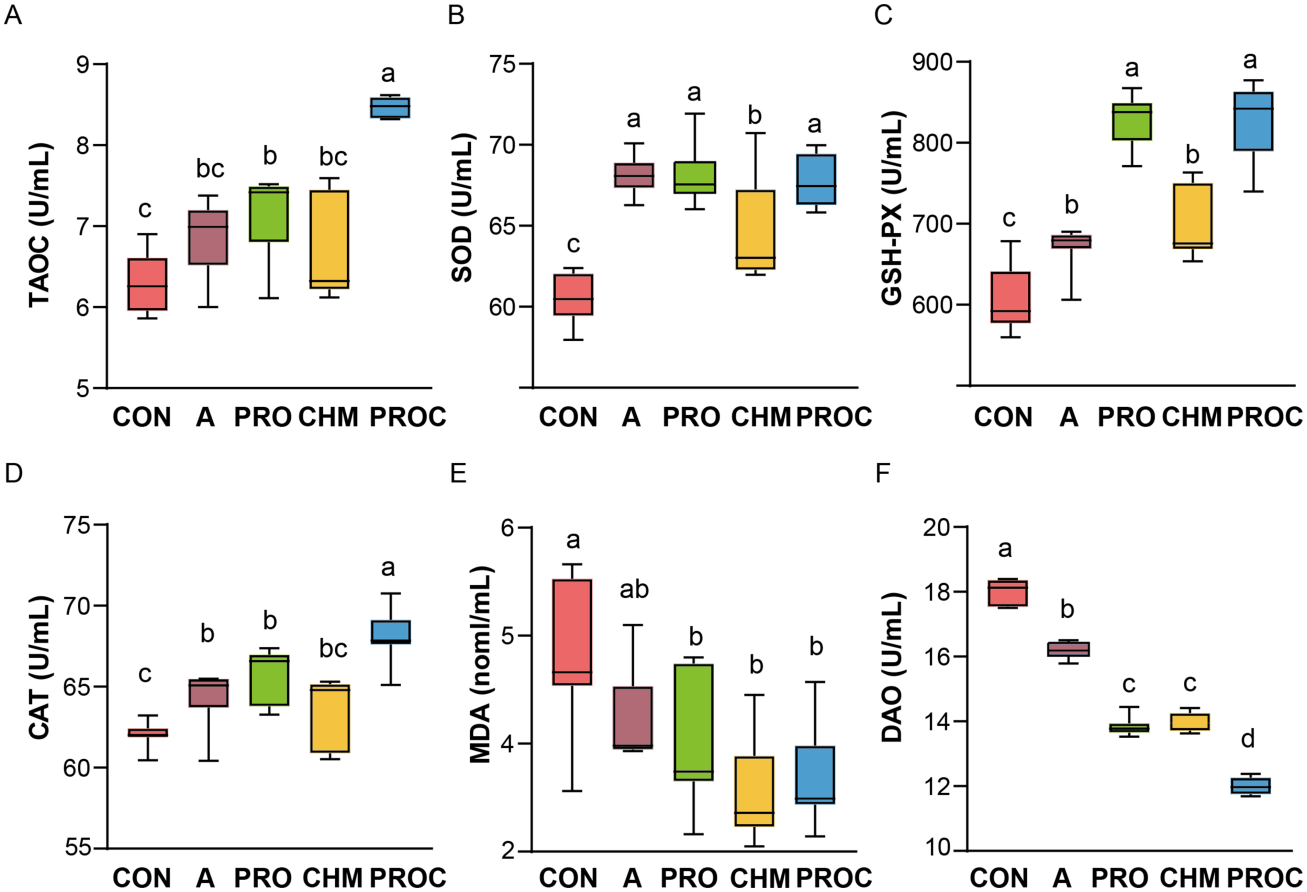
Effects of probiotics and/or Chinese herbal medicine on serum antioxidant parameters in weaned piglets. **(A)** T-AOC, **(B)** SOD, **(C)** GSH-Px, **(D)** CAT, **(E)** MDA, **(F)** DAO. Different letters indicate significant differences among groups (*p* < 0.05). Data are presented as mean ± SD (*n* = 9 per group). Different letters indicate significant differences among groups determined by one-way ANOVA followed by Tukey’s HSD post-hoc test for multiple comparisons (*p* < 0.05).

Serum antioxidant parameters (T-AOC, SOD, GSH-Px, CAT, DAO, and MDA) were measured after 28 days to assess oxidative stress status. The PROC group exhibited significantly higher T-AOC levels than all other groups (*p* < 0.05, Figure 2A). Compared with the control group, SOD levels increased by 12.30, 6.54, and 11.95% in the PRO, CHM, and PROC groups, respectively (Figure 2B). GSH-Px levels were markedly elevated in the PRO, CHM, and PROC groups relative to the CON group (*p* < 0.05, Figure 2C). Serum CAT levels were significantly higher in the PRO and PROC groups compared to the CON group (*p* < 0.05, Figure 2D). MDA levels were significantly reduced in the PRO, CHM, and PROC groups compared to the CON group (*p* < 0.05, Figure 2E). Serum DAO levels in the PROC group were the lowest compared to all treatment groups (Figure 2F).

### Effects of probiotics and Chinese herbal medicine on serum cytokines and immunoglobulins in weaned piglets

**Figure 3:**
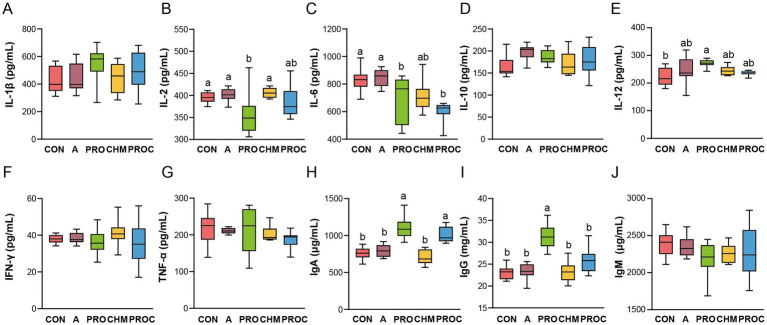
Determination of serum immune cytokines in different treatment groups. **(A)** IL-1β, **(B)** IL-2, **(C)** IL-6, **(D)** IL-10, **(E)** IL-12, **(F)** IFN-*γ*, **(G)** TNF-*α*, **(H)** IgA, **(I)** IgG, and **(J)** IgM. Data are presented as mean ± SD (*n* = 9 per group). Different letters indicate significant differences among groups determined by one-way ANOVA followed by Tukey’s HSD post-hoc test for multiple comparisons (*p* < 0.05).

Serum cytokine and immunoglobulin levels were measured to assess the inflammatory and immune status of weaned piglets. IL-2 levels in the PRO group were significantly reduced compared with the CON, A, and CHM groups (*p* < 0.05, Figure 3B). IL-6 concentrations in the PRO and PROC groups were significantly reduced compared with the CON and A groups, whereas the CHM group exhibited intermediate levels (*p* < 0.05, Figure 3C). IL-12 levels in the PRO group were markedly elevated compared to the CON group (*p* < 0.05, Figure 3E). Levels of IgA and IgG significantly increased in the PRO and PROC groups compared to the CON, A, and CHM groups (*p* < 0.05, Figures 3H,I). No significant differences were detected in the levels of IL-1β Figure 3A, IL-10 (Figure 3D), IFN-*γ*, (Figure 3F), TNF-*α*, (Figure 3G), and IgM (Figure 3J) among the five groups (*p* > 0.05).

### Effects of probiotics and Chinese herbal medicine on fecal microbiota composition and structure in weaned piglets

**Figure 4:**
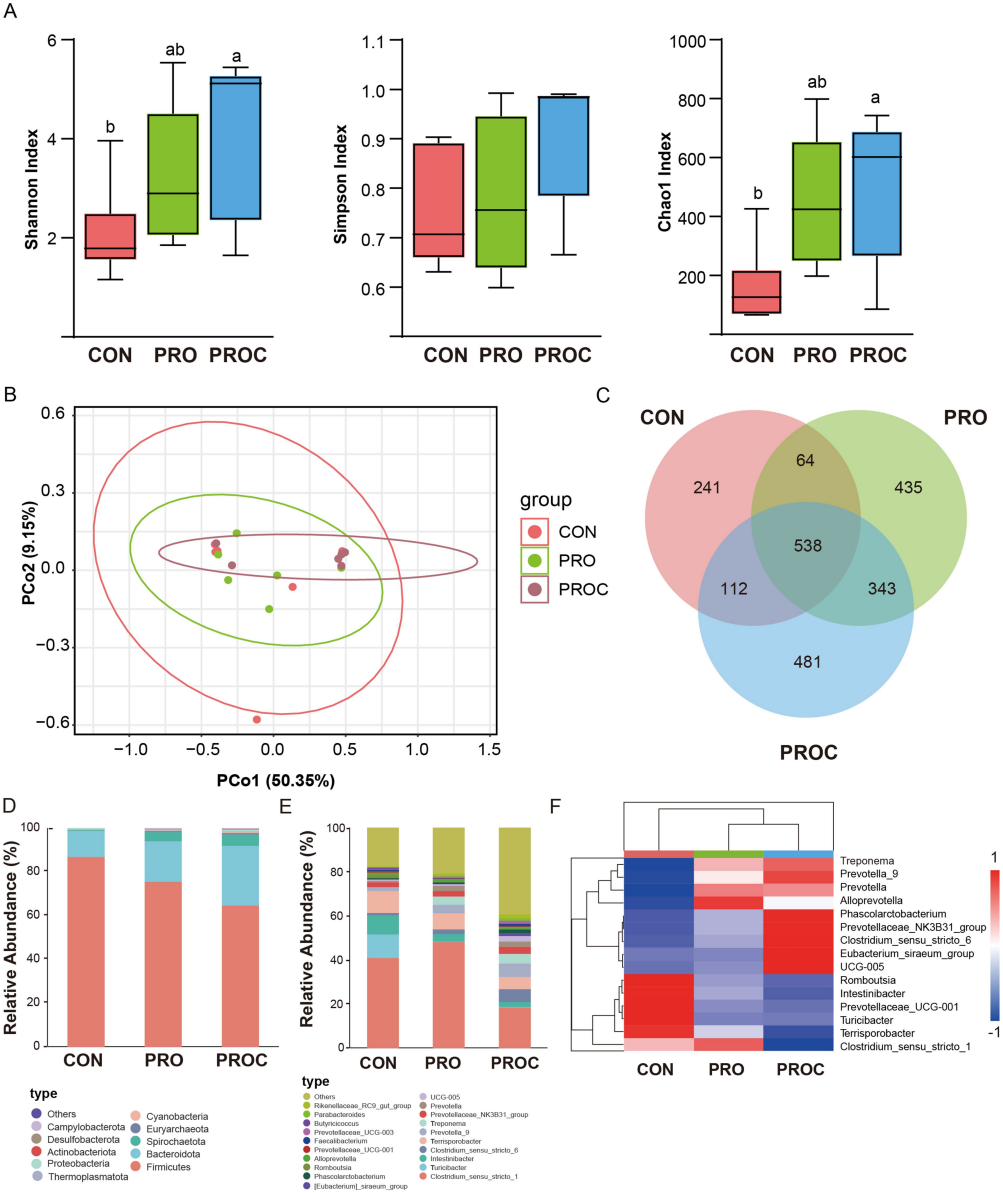
Microbiome diversity and composition analysis in weaned piglets feces. **(A)** Alpha diversity. Different letters indicate statistically significant differences (*p* < 0.05). **(B)** Beta diversity. **(C)** Venn diagram illustrating shared and unique ASVs (Amplicon Sequence Variants) among the three experimental groups. Taxonomic composition at phylum level **(D)** and genus level **(E)** presented as relative abundance percentages. **(F)** Heatmap visualization of differentially abundant taxa with hierarchical clustering.

Fecal microbiota composition analysis using 16S rRNA gene sequencing revealed that alpha diversity indices (Shannon, Simpson, and Chao1) progressively increased from the control group to the probiotic-treated groups (Figure 4A). The Shannon and Chao1 indices in the PROC group were significantly higher than those in the CON group (*p* < 0.05). Principal coordinate analysis (PCoA) demonstrated distinct clustering patterns among the three treatment groups (Figure 4B). This indicated that probiotic and Chinese herbal medicine supplementation induced significant shifts in fecal microbiota composition. The Venn diagram analysis identified 241, 435, and 481 unique ASVs for CON, PRO, and PROC groups, respectively (Figure 4C).

The fecal microbial composition at phylum (Top 10) and genus (Top 20) levels for different groups is presented in Figures 4D,E. At the phylum level, *Firmicutes*, *Bacteroidota*, and *Spirochaetota* were predominant across all groups. The PROC group exhibited an increased relative abundance of *Bacteroidota* and *Spirochaetota* compared to the control group. Microbial composition exhibited significant variation among treatments at the genus level. *Clostridium* sensu stricto 1 was predominant across all groups, exhibiting the highest abundance in the PRO group and a significant reduction in the PROC group. Conversely, PROC group showed enrichment of *Prevotella*_9 and *Clostridium*_sensu_stricto_6. Analysis of differential abundance identified two notable bacterial clusters (Figure 4F). The original cluster exhibited a markedly greater abundance in the CON group (*p* < 0.05), whereas the subsequent cluster was significantly enriched in both the PRO and PROC groups relative to the control.

**Figure 5:**
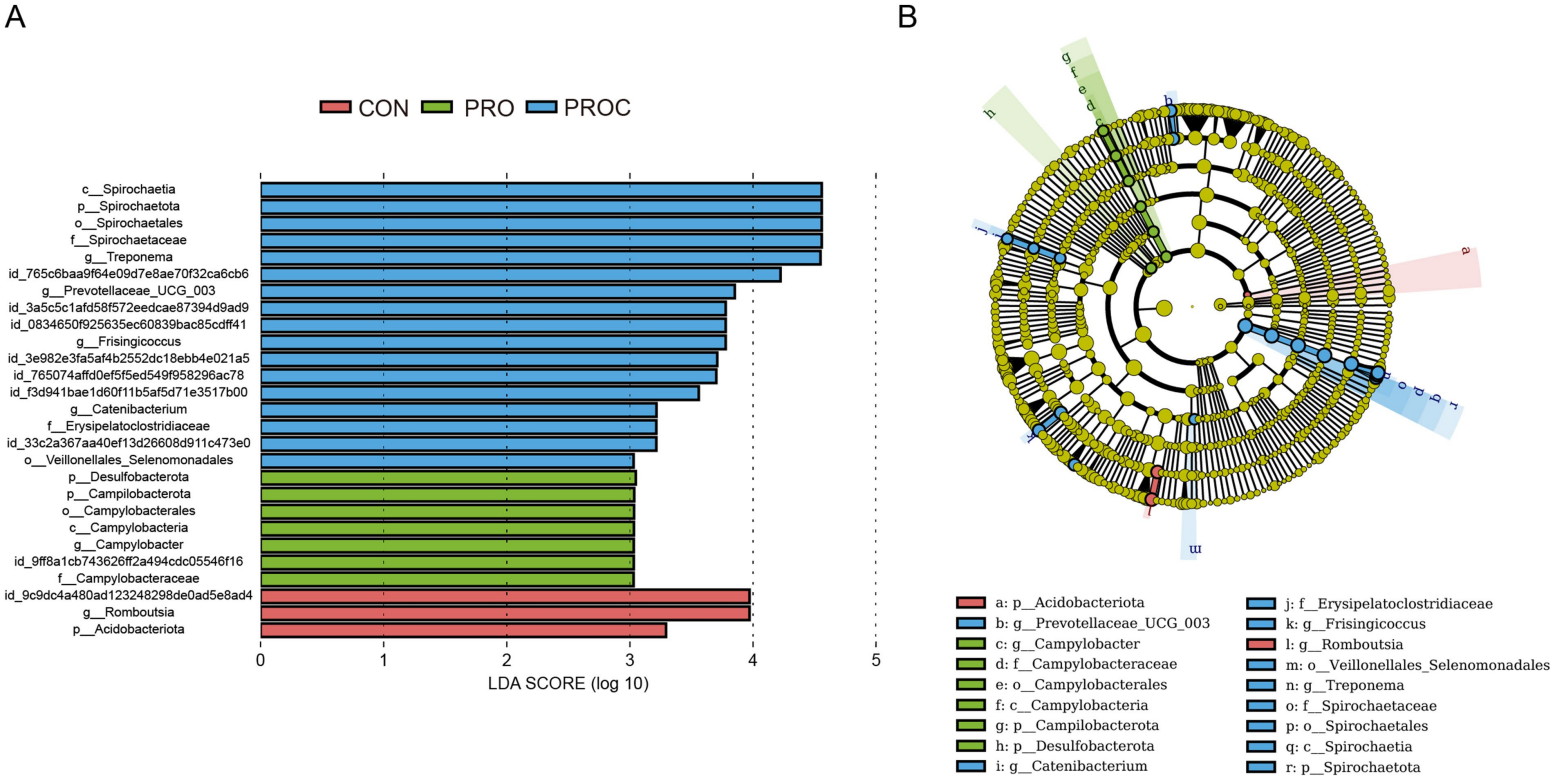
LEfSe analysis of fecal microbiota in weaned piglets. **(A)** LDA scores for significantly different taxa (LDA > 3, *p* < 0.05). **(B)** Cladogram showing enriched taxa by group (red: CON, green: PRO, blue: PROC). The tree structure shows taxonomic hierarchy from phylum to genus level.

LEfSe analysis identified taxonomic biomarkers with LDA scores >3 among the three groups (Figure 5). The CON group exhibited enrichment of two taxa (g_*Romboutsia,* p_*Acidobacteriota*), while the PRO group displayed six enriched taxa, predominantly related to *Campylobacter*. The PROC group included ten enriched taxa, comprising members of *Spirochaetota* and *Prevotellaceae*.

### Effects of probiotics and Chinese herbal medicine on fecal metabolome in weaned piglets

**Figure 6:**
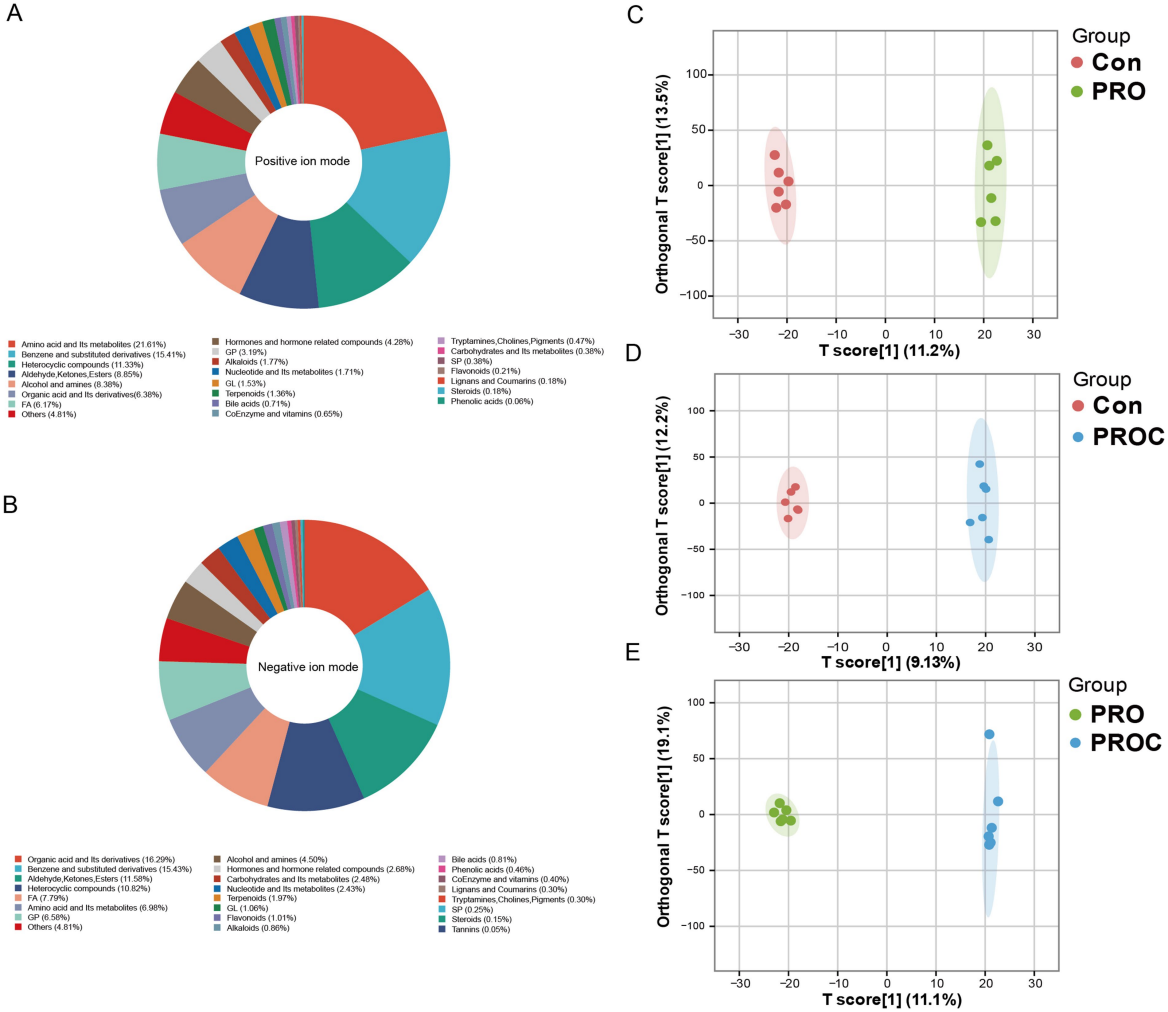
Metabolomics analysis of fecal samples from weaned piglets. Metabolite classification in **(A)** positive and **(B)** negative ion modes. **(C–E)** OPLS-DA score plots showing pairwise group comparisons.

Untargeted metabolomics identified 5,090 metabolites, with amino acids, benzene derivatives, and heterocyclic compounds being dominant (Figures 6A,B). OPLS-DA analysis demonstrated distinct metabolite profiles among treatment groups, highlighting significant metabolic differences (Figures 6C–E).

**Figure 7:**
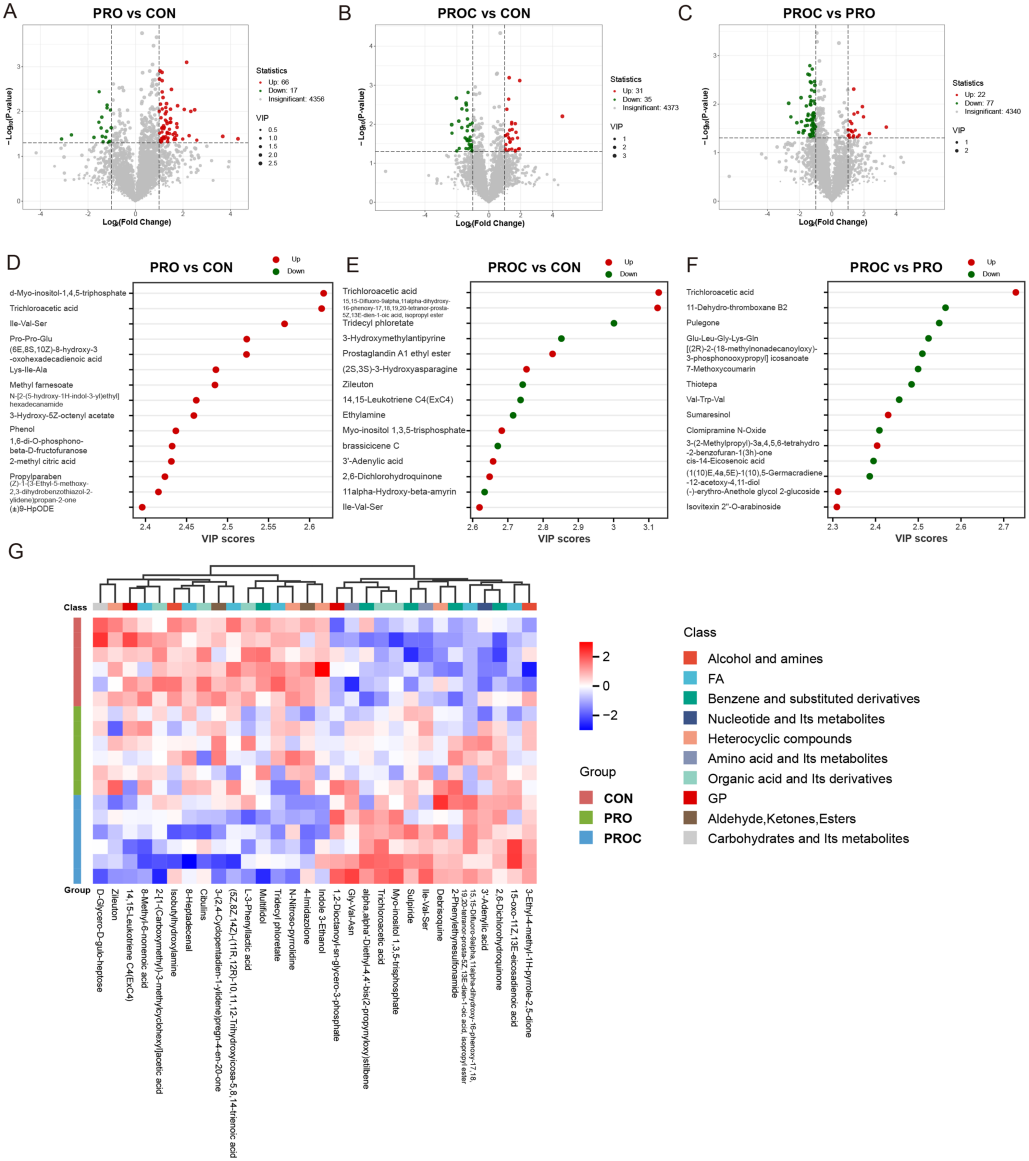
Differential fecal metabolite analysis among weaned piglet groups. **(A–C)** Volcano plots displaying metabolite changes between group comparisons. Red dots indicate upregulated metabolites, green dots show downregulated metabolites, and gray dots represent non-significant changes. Vertical dashed lines represent fold-change thresholds (log₂FC = ±1, equivalent to 2-fold change), and horizontal dashed lines represent *p*-value thresholds (*p* = 0.05, equivalent to -log₁₀*p* = 1.3). Metabolites above both thresholds are considered significantly differentially expressed. **(D–F)** VIP scores identifying key discriminatory metabolites for each comparison (VIP > 1). **(G)** Heatmap illustrating relative abundance patterns of differential metabolites across three groups. Color intensity reflects metabolite levels (red: high; blue: low).

Volcano plot analysis indicated notable metabolomic variations across the three treatment groups (Figures 7A–C). In the comparison of PRO vs. CON, 56 metabolites were significantly upregulated and 17 were downregulated (Figure 7A). The PROC vs. CON comparison identified 31 upregulated and 35 downregulated metabolites (Figure 7B). When comparing PROC with PRO groups, 22 metabolites showed upregulation while 77 exhibited downregulation (Figure 7C).

The VIP analysis identified the top 15 most discriminatory metabolites for each comparison (Figures 7D–F). The PRO group exhibited higher concentrations of organic acids, fatty acids, amino acid derivatives, and benzene compounds compared to the CON group (Figure 7D). The comparison between PROC with CON demonstrated intricate metabolic changes (Figure 7E). Notable upregulated metabolites comprised trichloroacetic acid, prostaglandin derivatives, and amino acid metabolites. Metabolites that were downregulated mainly included phenolic compounds and lipid derivatives. In the comparison of PROC and PRO, metabolites with high VIP scores showed distinct regulation patterns (Figure 7F). Upregulated metabolites included organic acids and plant-derived compounds, while downregulated metabolites encompassed lipid metabolites and derivatives of amino acids.

Hierarchical clustering analysis demonstrated clear metabolic profile separation among the three groups (Figure 7G). The heatmap demonstrated clear patterns of metabolite expression, categorising metabolites into functional classes such as alcohols and amines, fatty acids, benzene derivatives, nucleotides, heterocyclic compounds, amino acids, organic acids, and carbohydrates. The CON group demonstrated a distinct expression pattern compared to both treatment groups, whereas the PRO and PROC groups exhibited partially overlapping yet distinguishable metabolic signatures.

**Figure 8:**
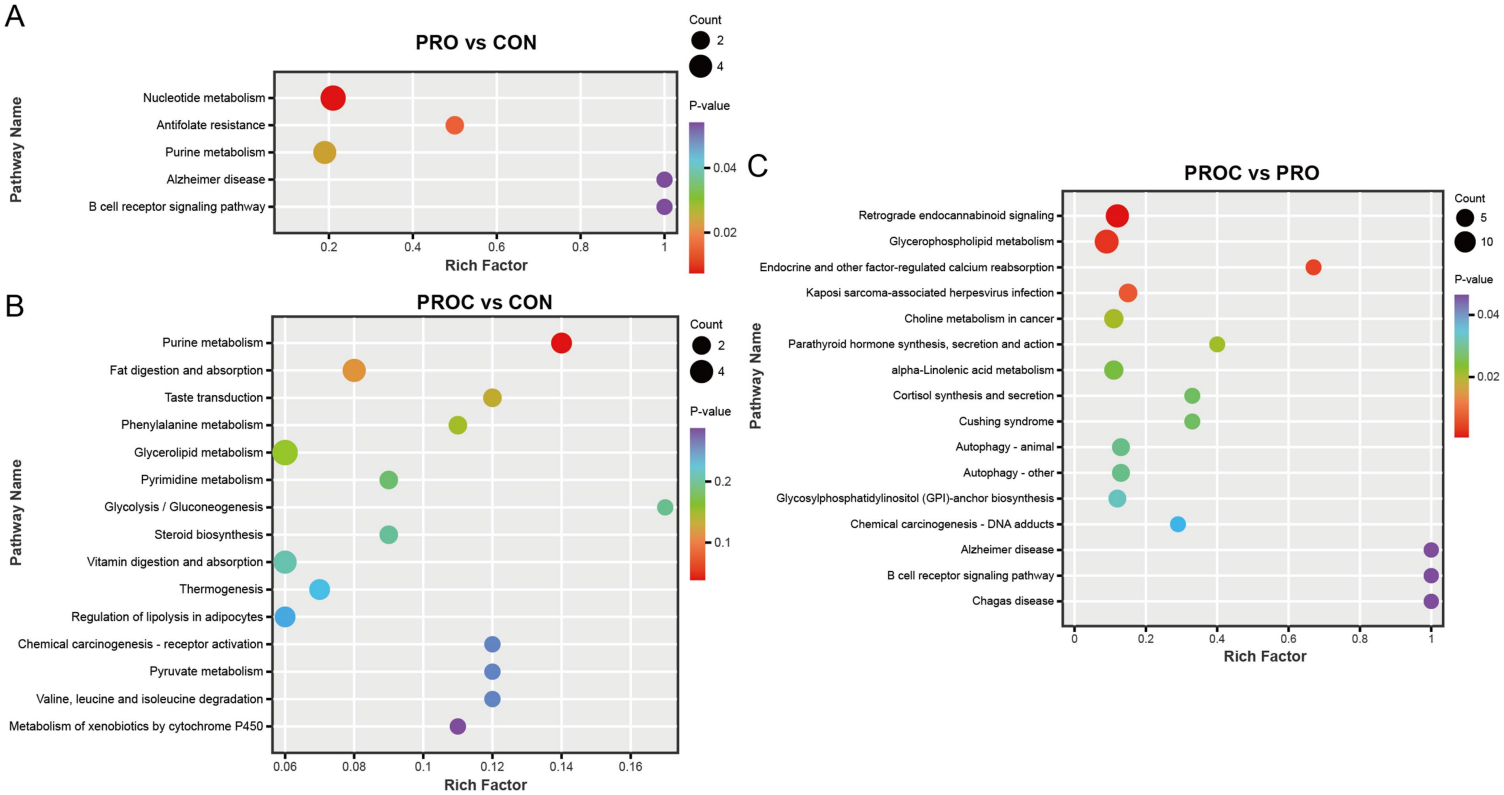
KEGG pathway enrichment analysis of differential fecal metabolites in weaned piglets. Bubble plots show significantly enriched pathways (*p* < 0.05) for **(A)** PRO vs. CON, **(B)** PROC vs. CON, and **(C)** PROC vs. PRO comparisons. Bubble size represents metabolite count in each pathway, while color intensity indicates *p*-value significance.

KEGG pathway enrichment analysis showed that probiotic and probiotic-herbal treatments significantly altered the distributions of fecal metabolite pathways in weaned piglets (Figure 8). The PRO group exhibited enrichment in five pathways relative to the CON group. Nucleotide metabolism and antifolate resistance pathways exhibited the highest statistical significance, succeeded by purine metabolism (Figure 8A).

The PROC group affected more metabolic pathways than the CON group (Figure 8B). These encompassed metabolic pathways including purine metabolism, fat digestion and absorption, and glycerolipid metabolism. In the comparison of PROC and PRO group, retrograde endocannabinoid signaling showed the highest enrichment factor. This comparison also revealed significant enrichment in glycerophospholipid metabolism, endocrine and other factor-regulated calcium reabsorption, and Cushing syndrome pathways.

### Correlation analysis between microbiota and metabolites in weaned piglets feces

**Figure 9:**
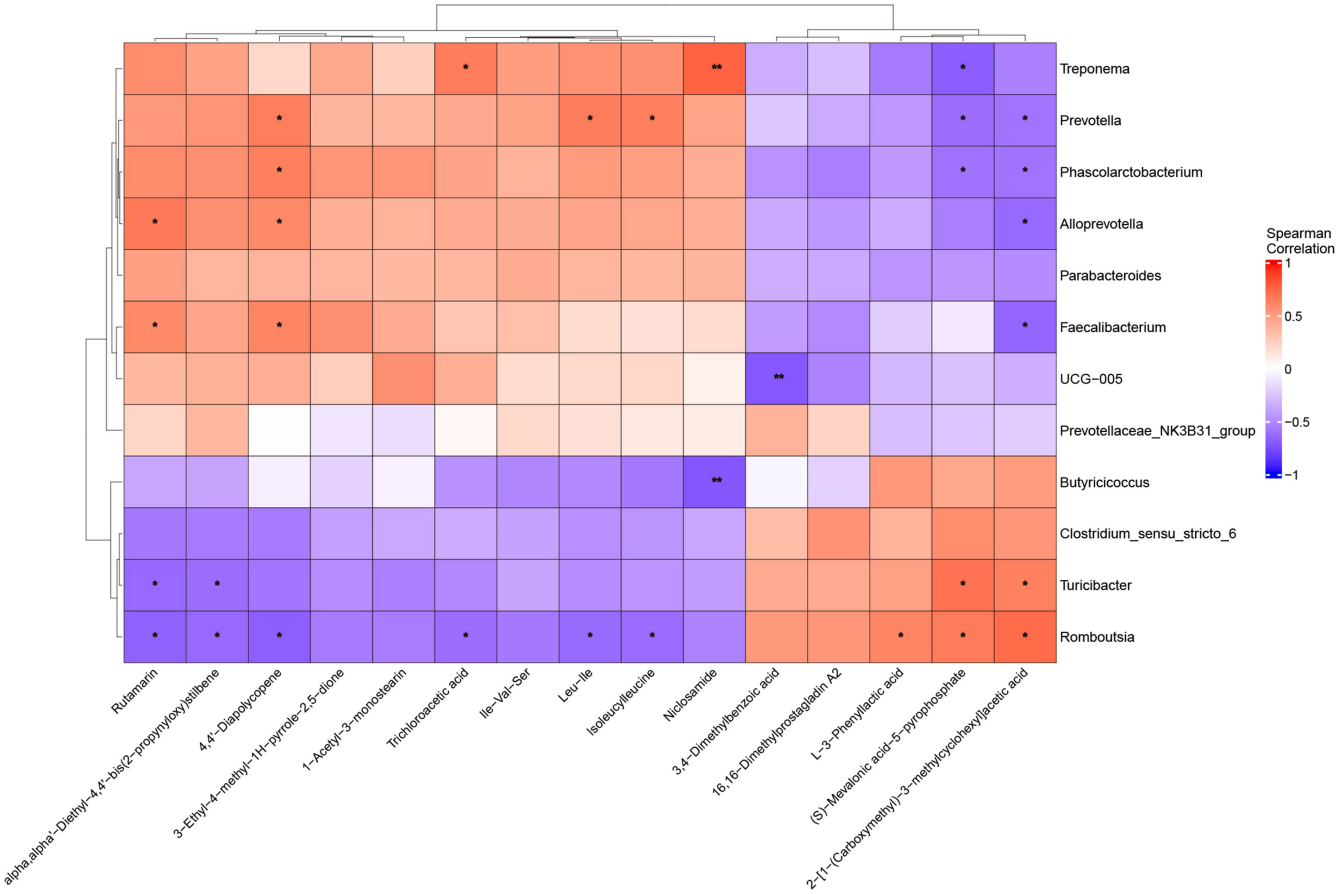
Spearman correlation analysis between fecal microbiota and metabolites in weaned piglets. Red colors indicate positive correlations, while blue colors represent negative correlations, with color intensity reflecting correlation strength (scale: −1 to +1). *: *p* < 0.05, **: *p* < 0.005.

Spearman correlation analysis revealed associations between microbiota and metabolites in the feces of weaned piglets (Figure 9). Among these differential metabolites, Treponema showed significant positive correlations with Niclosamide (*p* < 0.005) and Trichloroacetic acid (*p* < 0.05), while demonstrating a negative correlation with (S)-Mevalonic acid-5-pyrophosphate (*p* < 0.05). Prevotella displayed positive correlations with 4,4′-Diapolycopene, Isoleucylleucine, and Leu-Ile, but negative correlations with (S)-Mevalonic acid-5-pyrophosphate and 2-[1-(Carboxymethyl)-3-methylcyclohexyl]acetic acid (*p* < 0.05). Turicibacter showed positive correlations with 2-[1-(Carboxymethyl)-3-methylcyclohexyl]acetic acid and (S)-Mevalonic acid-5-pyrophosphate, while showing correlations with alpha, alpha′-Diethyl-4,4′-bis(2-propynyloxy)stilbene and Rutamarin (*p* < 0.05).

## Discussion

Weaning stress constitutes a major challenge in modern pig production, causing considerable economic losses through reduced growth performance, increased disease susceptibility, and higher mortality rates (31, 33–35). The transition from maternal milk to solid feed triggers complex physiological and immunological changes that disrupt intestinal homeostasis and compromise animal welfare (36, 37). These complications highlight the urgent need for effective intervention strategies.

Previous research has established beneficial effects of single-strain probiotics and individual Chinese herbal medicines in weaned piglets. However, these approaches show limited efficacy compared with integrated intervention strategies. For example, *Bacillus* species can prevent diarrhea and improve growth performance in weaned piglets (38). *Lactobacillus sali*var*ius* has shown promise in enhancing immune function in weaned piglets (39). Chinese herbal medicines have also demonstrated positive effects on piglet health through immune modulation and antioxidant enhancement. This immunomodulatory potential is exemplified by *Magnolia officinali*s extract, which effectively modulated immune responses and reduced diarrhea in weaned piglets through enhanced anti-inflammatory cytokine expression and improved gut health indicators (40). Plant-derived compounds, such as essential oils and herbal extracts, have shown promise in enhancing immune responses and modulating gut microbiota, thereby improving growth and health in pigs (41). However, the efficacy of Chinese herbal medicine depends not only on its bioactive components but also on their absorption and transformation, as pharmacokinetic studies highlight variable bioavailability of herbal constituents in animal models (42). This variability in bioavailability represents a significant challenge when using herbal interventions as standalone treatments. This challenge is exemplified in our study, where the CHM group’s increased feed consumption (6.35% higher ADFI) without proportional weight gain suggests reduced feed conversion efficiency, possibly due to anti-nutritional factors in herbal compounds and limited bioavailability without microbial enhancement (43). Most studies have focused on single interventions, with limited investigation of multi-strain probiotic and herbal combinations at the molecular level (44).

This study aimed to overcome existing limitations by formulating a comprehensive intervention strategy that integrates five probiotic strains with three traditional Chinese herbs. The synergistic effects were systematically evaluated using various methodologies, including growth performance assessment, serum biochemical analysis, and multi-omics approaches.

Fermentation product characterization demonstrated clear advantages of multi-strain over single-strain systems, including enhanced pH reduction, increased organic acid production, and higher protein content. Enhanced organic acid production, particularly butyric acid (increasing from 0.613–1.323 mg/mL in single strains to 1.613 mg/mL in mixed culture), indicates complex metabolic complementarity among strains. *Lactobacillus plantarum* and *P. pentosaceus* likely provide lactic acid as substrate for *B. coagulans* and *S. boulardii* to produce butyric acid through cross-feeding mechanisms, where metabolic products from one strain serve as nutrients for another (45). The increase in crude protein suggests improved proteolytic activity, likely due to complementary enzyme systems in which peptidases from various strains collaborate to break down complex proteins into bioactive peptides (46). The lower pH (3.887 vs. 4.077–4.343) and enhanced organic acid production in mixed fermentation indicate improved antimicrobial potential and intestinal acidification capacity (47). In Chinese herbal medicine formulations, Codonopsis total saponins provide antioxidant effects, boost immunoglobulin levels, and reduce inflammation (48). The main active compounds in *Astragalus* are saponins, polysaccharides, and flavonoids. These compounds have shown anti-inflammatory, antioxidant, and anti-apoptotic activities (49–51). The main components of *Magnolia officinalis* are magnolol and honokiol. *Magnolia* extract can reduce lipid oxidation in finishing pigs (52). We used post-fermentation mixing rather than co-fermentation to avoid potential inhibitory effects. Post-fermentation mixing was employed instead of co-fermentation for two primary reasons. This approach reduces the inhibitory effects of tannins, alkaloids, and other compounds present in Chinese herbal medicine on probiotic fermentation (53, 54). Additionally, it prevents possible degradation or structural alterations of active ingredients throughout the fermentation process.

Both probiotic treatments significantly improved growth performance, with the combined treatment (PROC) achieving optimal results. The 8.91% increase in final body weight and feed conversion ratio of 1.55 represent substantial improvements over conventional approaches. Weaning induces substantial free radical production and oxidative stress in piglets, making antioxidant capacity crucial for maintaining health (55). Additionally, dietary and environmental transitions cause diarrhea, intestinal barrier damage, and gut inflammation in weaned piglets (56, 57).

This study shows that combined supplementation of probiotics and herbal formula in weaned piglet diets significantly increased serum T-AOC, SOD, GSH-PX, and CAT levels, while inflammatory cytokines IL-2 and IL-6 decreased notably. These improvements indicate effective mitigation of weaning-induced oxidative stress and inflammatory responses. The immunoglobulin response pattern suggests balanced immune modulation rather than simple stimulation (58). While serum IgA and IgG levels in the PRO group were significantly higher than in controls, the PROC group showed moderate reductions compared to the PRO group. The PROC group showed moderate decreases in IgA and IgG levels compared to the PRO group. However, both treatment groups remained significantly higher than controls (*p* < 0.05). This pattern suggests that herbal components modulate rather than simply stimulate humoral immunity (59). Given that growth performance and other health indicators were maintained or improved in the PROC group, this change likely reflects immune system optimization rather than impairment. However, the precise mechanisms underlying this immunoglobulin pattern require further investigation through targeted immune pathway analysis.

The microbiota analysis revealed significant community restructuring that supports improved intestinal health. Gut microbes and their metabolites play important roles in maintaining intestinal barrier function and gut environmental homeostasis. Recent studies have shown that supplementation with live and inactivated *Akkermansia muciniphila* improved inflammatory responses and enhanced antioxidant capacity in weaned piglets, while also altering microbial community composition in the ileum and colon (60). Fermented feed containing *Lactobacillus plantarum* and *Bacillus subtilis* improved gut microbiota structure and metabolic profiles in weaned piglets, and also regulated metabolic pathways of tryptophan, phenylalanine, and steroid hormone biosynthesis (61). This study found that multi-strain probiotics and Chinese herbal medicine as feed additives for weaned piglets significantly increased *α* diversity of fecal microbial communities. Based on *β*-diversity results, the PROC group showed a separation trend compared with the CON group, indicating that PROC treatment had significant regulatory effects on community structure. The distinct clustering pattern demonstrates successful ecological intervention. Our findings that combined probiotic and herbal supplementation promoted intestinal development and improved microbial balance are consistent with recent evidence that hydrolyzed protein-based diets can enhance gut morphology and alter cecal microbiota in low-birth-weight piglets (62). This altered the original community composition patterns and resulted in diversity characteristics that differed from the CON group. At the phylum level, *Firmicutes* and *Bacteroidetes* were the main dominant groups. The PRO and PROC groups showed decreased ratios of *Firmicutes* to *Bacteroidetes*. Previous studies indicated that higher F/B ratios correlated with increased probability of diarrhea or illness in animals (63, 64). The enrichment of beneficial bacteria with specific functions represents a key mechanism of action. LEfSe analysis showed that the PROC group enriched *Spirochaetota* bacteria with cellulose and plant polysaccharide degradation ability (65), as well as Prevotellaceae_UCG_003 that produces short-chain fatty acids (66). This indicated that synergistic effects of probiotics and Chinese herbal medicine not only changed fecal microbiota composition, but also potentially improved gut health status of weaned piglets through promoting digestion and absorption, producing short-chain fatty acids, and enhancing intestinal barrier function.

The metabolomic analysis provided molecular-level evidence for the beneficial effects observed at the phenotypic level. Based on fecal untargeted metabolomics results, total metabolite detection showed rich metabolite profiles in both positive and negative ion modes. These mainly included amino acids and their metabolites, hormones and hormone-related compounds, lipids, organic acid derivatives, flavonoid compounds and other metabolic categories. OPLS-DA analysis showed that both PRO group and PROC group displayed clear separation from the control group in metabolic profiles. The two treatment groups also showed significant separation from each other. These results indicated that multi-strain probiotics used alone or combined with Chinese herbal medicine formulas both significantly changed the gut metabolic status of weaned piglets.

Key metabolite changes reflect enhanced cellular signaling and anti-inflammatory responses. VIP score analysis identified key metabolites that distinguished between different interventions. Compared to the CON group, the PRO group significantly upregulated key signaling molecule *α*-Myo-inositol-1,4,5-triphosphate, amino acid metabolites (Ile-Val-Ser, Pro-Pro-Glu, Lys-Ile-Ala) and organic acids. These findings were consistent with previous reports that probiotics could regulate host amino acid metabolism and cell signaling pathways (67). Amino acid nutrition and microbial modulation are increasingly recognized as critical for maintaining intestinal health, with evidence from ruminant studies suggesting that dietary strategies improving amino acid balance can influence gut microbial composition (68). Compared to the CON group, the PROC group showed different metabolic patterns, characterized by significantly enhanced anti-inflammatory activity. Notably, the significant downregulation of 14,15-Leukotriene C4 and upregulation of Prostaglandin A1 ethyl ester indicated a shift toward an anti-inflammatory environment. 14,15-Leukotriene C4 is a potent pro-inflammatory mediator involved in various inflammatory diseases (69), while prostaglandin compounds can exert protective effects on gastric mucosa and intestinal epithelium (70). Interestingly, Zileuton was also downregulated. As a specific inhibitor of 5-lipoxygenase (5-LOX), Zileuton can block the conversion pathway from arachidonic acid to leukotrienes. This suggests the establishment of a new inflammatory-anti-inflammatory equilibrium. The upregulation of plant-derived antioxidants confirms the contribution of herbal components. Direct comparison between PROC and PRO groups revealed specific metabolic contributions of Chinese herbal medicine components. Significant upregulation of plant-derived antioxidant compounds, including lignin compound Sumaresinol and flavonoid glycoside Isovitexin 2”-O-arabinoside, demonstrated enhanced antioxidant capacity achieved by Chinese herbal medicine supplementation (71, 72).

KEGG pathway enrichment analysis provided systematic molecular mechanism explanations for the observed differential metabolite changes. The analysis revealed hierarchical synergistic regulatory patterns of the probiotic-Chinese herbal medicine intervention. The persistent significant enrichment of Nucleotide metabolism and Purine metabolism in the PRO group directly corresponded to upregulation of 3′-adenosine monophosphate, indicating that nucleotide metabolism was an important target of probiotic intervention and provided fundamental support for cellular energy metabolism and nucleic acid synthesis. Significant enrichment of lipid metabolism pathways including Glycerolipid metabolism and Fat digestion and absorption in the PROC group indicated systematic enhancement of lipid metabolism, providing molecular basis for short-chain fatty acid generation and lipid signal regulation. The observed enrichment of metabolic pathways, particularly those related to purine and lipid metabolism, may be linked to microbial-derived metabolites such as butyrate, which are known to regulate host cellular functions and inflammatory signaling (73). This connection between microbial metabolism and host physiological regulation provides additional mechanistic insight into how probiotic interventions modulate cellular energy metabolism and immune responses in weaned piglets. The activation of autophagy pathways specifically in the PROC group suggests additional cellular protective mechanisms (74). Particularly noteworthy was the specific enrichment of Autophagy—animal pathway in the PROC group, which reflected unique contributions of Chinese herbal medicine components. Autophagy activation is crucial for intestinal epithelial cell homeostasis during weaning stress, as it facilitates the removal of damaged organelles and proteins while promoting cellular renovation (75, 76).

The correlation analysis revealed functional relationships between specific bacterial taxa and key metabolites. Spearman correlation analysis between microbiota and metabolome revealed functional association networks between gut microbes and key metabolites. *Treponema* showed specific metabolic association patterns in the gut of weaned piglets. This genus showed significant positive correlation with Niclosamide (*p* < 0.05). Niclosamide can disrupt mitochondrial metabolism of tapeworms. Additionally, Niclosamide was proven to achieve anti-infection effects by inhibiting production of acyl-homoserine lactones (a quorum sensing signal molecule), thereby preventing *Pseudomonas aeruginosa* quorum sensing responses (77). Niclosamide has also been proven to have broad-spectrum antiviral activity (78). Prevotella showed significant positive correlations with 4,4’-Diapolycopene, Isoleucylleucine and Leu-Ile (*p* < 0.05). These associations suggest roles in both antioxidant metabolism and protein utilization. As a precursor substance for carotenoid synthesis, the enrichment of 4,4’-Diapolycopene revealed that *Prevotella* may participate in regulation of antioxidant metabolic networks (79). Meanwhile, the positive correlations between this bacterium and Isoleucylleucine and Leu-Ile indicated its potential positive role in protein metabolism and muscle protein synthesis, which is crucial for growth and development of weaned piglets (80).

Several limitations should be acknowledged. Due to resource constraints, microbiome and metabolomic analyses were limited to the two most promising treatment groups (PRO and PROC) based on their superior performance in growth, antioxidant capacity, and inflammatory responses. Additionally, the 28-day experimental period may not capture long-term effects or sustained benefits that are crucial for commercial application. The use of a single pig genotype (Duroc × Landrace × Yorkshire) may limit the generalizability of our findings across different genetic backgrounds. Our study did not test different dosages of probiotics and herbal compounds on piglet growth and intestinal development. Future research should examine dose–response relationships and investigate the persistence of beneficial effects. We plan to examine the cooperative effects between different bacterial strains in the probiotic blend. This includes studying how these bacteria establish themselves in the piglet intestine, which metabolites they generate, and their ability to control pathogenic microbes.

## Conclusion

In conclusion, combined supplementation with fermented multi-strain probiotics solutions and Chinese herbal medicine effectively improved growth performance, serum antioxidant and immune parameters, and fecal microbiota composition and metabolite profiles in weaned piglets, with the PROC group showing the most significant improvements. The combined intervention increased gut microbial *α*-diversity and improved microbiota structure, creating a unique metabolic reprogramming pattern. Furthermore, correlation analysis between microbiome and metabolome data revealed functional networks linking specific bacterial species with key metabolites involved in antioxidant defense, immune regulation, and protein metabolism. Our findings demonstrate that the probiotic-herbal combination created synergistic effects that enhanced gut health and development in weaned piglets, providing an effective nutritional intervention strategy for weaning stress management. However, this study was limited by the 28-day experimental period, single pig genotype, and lack of dose–response investigation, which may affect the generalizability and optimization of these interventions. Future research should focus on long-term efficacy studies, multi-breed validation, dose–response optimization, and detailed mechanistic investigations to further validate and enhance the practical application of probiotic-herbal synergistic effects in commercial swine production.

## Data Availability

The sequence data have been deposited in the NCBI Sequence Read Archive (SRA) under accession number PRJNA1330057. The original contributions presented in the study are included in the article/supplementary material. Further inquiries can be directed to the corresponding author.
